# Retinal vascular occlusion in patients with the Covid-19 virus

**DOI:** 10.1186/s40942-022-00371-7

**Published:** 2022-06-23

**Authors:** Helio F. Shiroma, Luiz H. Lima, Yuri B. Shiroma, Tereza C. Kanadani, Mario J. Nobrega, Gabriel Andrade, Milton Nunes de Moraes Filho, Fernando M. Penha

**Affiliations:** 1 Hospital Infantil Joana de Gusmão, Rua Rui Barbosa 152, Florianopolis (SC), ZIP: 88025-301 Brazil; 2 Universidade Federal de Sao Paulo, Rua Botucatu 822, Sao Paulo (SP), ZIP: 04023-062 Brazil; 3 Pontificia Universidade Catolica Do Parana, Rua Imaculada Conceicao 1155, Curitiba (PR), ZIP: 80215-901 Brazil; 4 Universidade Federal de Minas Gerais, Rua Antonio Carlos 6627, Belo Horizonte (MG), ZIP: 31270-901 Brazil; 5 Hospital Sadalla Amin Ghanem, Rua Camboriu 35, Joinville (SC), ZIP: 89216-222 Brazil; 6 Instituto de Olhos Colatina, Rua Aroldo Antolini 127, Colatina (ES), ZIP: 29702-080 Brazil; 7 Fundacao Universidade Regional de Blumenau, Rua Antonio Veiga 140, Blumenau (SC), ZIP: 89030-903 Brazil; 8 Hospital Infantil Joana de Gusmão, Rua William Richard Schisler 900 apto 622, 88.034-100 Florianopolis (SC), Brazil

**Keywords:** Retina, Vascular occlusion, COVID-19, Thromboembolic phenomena, Intravitreal injections

## Abstract

**Background:**

The coronavirus disease (COVID-19) can cause acute respiratory distress syndrome with dyspnea, anosmia, fever, and cough. Few studies describing ocular findings have been reported. The current case series, reports the clinical findings and natural history of patients with retinal vascular occlusion after COVID-19 infection.

**Case presentations:**

Patients from multiple Brazilian hospitals who had clinical and laboratory diagnoses of COVID-19 with retinal vein or arterial occlusion were analyzed retrospectively. The baseline demographics, clinical presentations of COVID-19, comorbidities, risk factors for thromboembolic events, and use of anticoagulant drugs were reviewed. The relevant clinical findings associated with the retinal vascular occlusive event, management, and outcomes were reported. Fourteen cases of retinal vascular occlusion within 3 months of the laboratory confirmed COVID-19 infection were identified. Three of which required hospitalization for COVID-19 management. Eight cases had central retinal vein occlusion, three branch retinal vein occlusion, one hemispheric retinal vein occlusion, and two central arterial occlusion. The mean patient age at presentation was 48 years; the visual acuity ranged from light perception to 20/20. Nine patients received intravitreal injections of anti-angiogenic drugs and one received ketorolac tromethamine drops for the management of secondary macular edema; four were untreated.

**Conclusions:**

COVID-19 patients may rarely have ocular manifestations of the disease. It was presented a case series of vascular occlusion events that may be related to COVID-19 infection, since these thrombotic events are actively involved in the disease pathophysiology. These cases emphasize the need for further investigation of ocular complications associated with this disease.

## Background

An outbreak of coronavirus disease (COVID-19) was first reported as a pneumonia case in Wuhan, China, in December 2019 [1], and spread quickly worldwide. COVID-19, officially named severe acute respiratory syndrome coronavirus 2 (SARS-CoV-2), is a single-stranded RNA virus. Most patients are either asymptomatic or have mild symptoms. However, disease progression might lead to acute respiratory distress syndrome and even death [2].

The most common symptoms of COVID-19 include dyspnea, anosmia, fever, and cough. The ocular findings described in the literature include conjunctivitis [3], intraocular inflammation [4], and retinal changes on optical coherence tomography (OCT), with hyperreflective lesions at the level of the ganglion cell and inner plexiform layers [5].

Although few cases of retinal vascular occlusion in patients with COVID-19 have been reported in the literature, the explanation for such an association is unknown. We report 14 patients with retinal vascular occlusion. The current study both reports a unique and large series of retinal vascular occlusion cases, including arterial and venous vessels, in relatively young patients with COVID-19 and forwards some hypotheses for this occurrence [6].

## Case presentation

The data were collected retrospectively from multiple Brazilian centers from public and private hospitals, by members of the Brazilian Retina and Vitreous Society. The present study was approved by the Research Ethics Committee of the Fundacao Universidade Regional de Blumenau (protocol number, 38762920.2.0000.5370).

Data were collected regarding the treatment of COVID, the presence of retinal vein or arterial occlusion, included reverse transcriptase-polymerase chain reaction (RT-PCR) positivity, age, sex, comorbidities, initial and final visual acuities (VAs), hospitalization, necessity for anticoagulant drugs, time after infection when the VA decreased, and the treatments of the occlusions administered, i.e., none, anti-inflammatory non-hormonal drops, antivascular endothelial growth factor (VEGF) injections or dexamethasone intravitreal implant. Patients were excluded who had previous decompensated hypertension, diabetes, thromboembolic phenomena, and cardiovascular disease.

All data from patients in whom a retinal vascular occlusion developed after COVID-19 were collected between August 15, 2020, and October 30, 2020, after the patients provided informed consent.

The patients were divided into four groups: 1, those with central retinal vein occlusion (CRVO); 2, those with hemispheric retinal vein occlusion (HRVO); 3, those with branch retinal vein occlusion (BRVO); and 4, those with central retinal arterial occlusion (CRAO).

## Results

A total of 14 cases of retinal vascular occlusion following COVID-19 infections that were confirmed by RT-PCR were identified. The mean patient age was 47.97 years and most patients (85.72%) had no previous history of systemic hypertension or thrombotic events.The severity of the retinal vascular occlusive events in the present case series varied from mild retinal vein occlusion with a BCVA exceeding 20/60 in eight cases, severe retinal vein occlusion with a BCVA worse than 20/60 in three cases, and retinal arterial occlusion in 2 cases.

## Group 1

Eight patients (6 men, 2 women; average age, 43.1 years; range, 27 to 65) with CRVO were identified. Five patients had a VA of 20/40 better and four had a VA below 20/40 (range, 20/20–20/1000). Patient 5, was an emblematic case. No previous medical history, with 27 years old who presented with CRVO had D-dimer of 1232 ng/mL and PCR of 1,9 mg/L, the remain laboratory workup was unremarkable. Protein C, S and antiphospholipid antibody were normal. Six patients were treated with intravitreal anti-VEGF drugs. Two patients with a VA of 20/20 were followed clinically. The VA improved in all treated patients; three patients achieved 20/20.

## Group 2

**Fig. 1:**
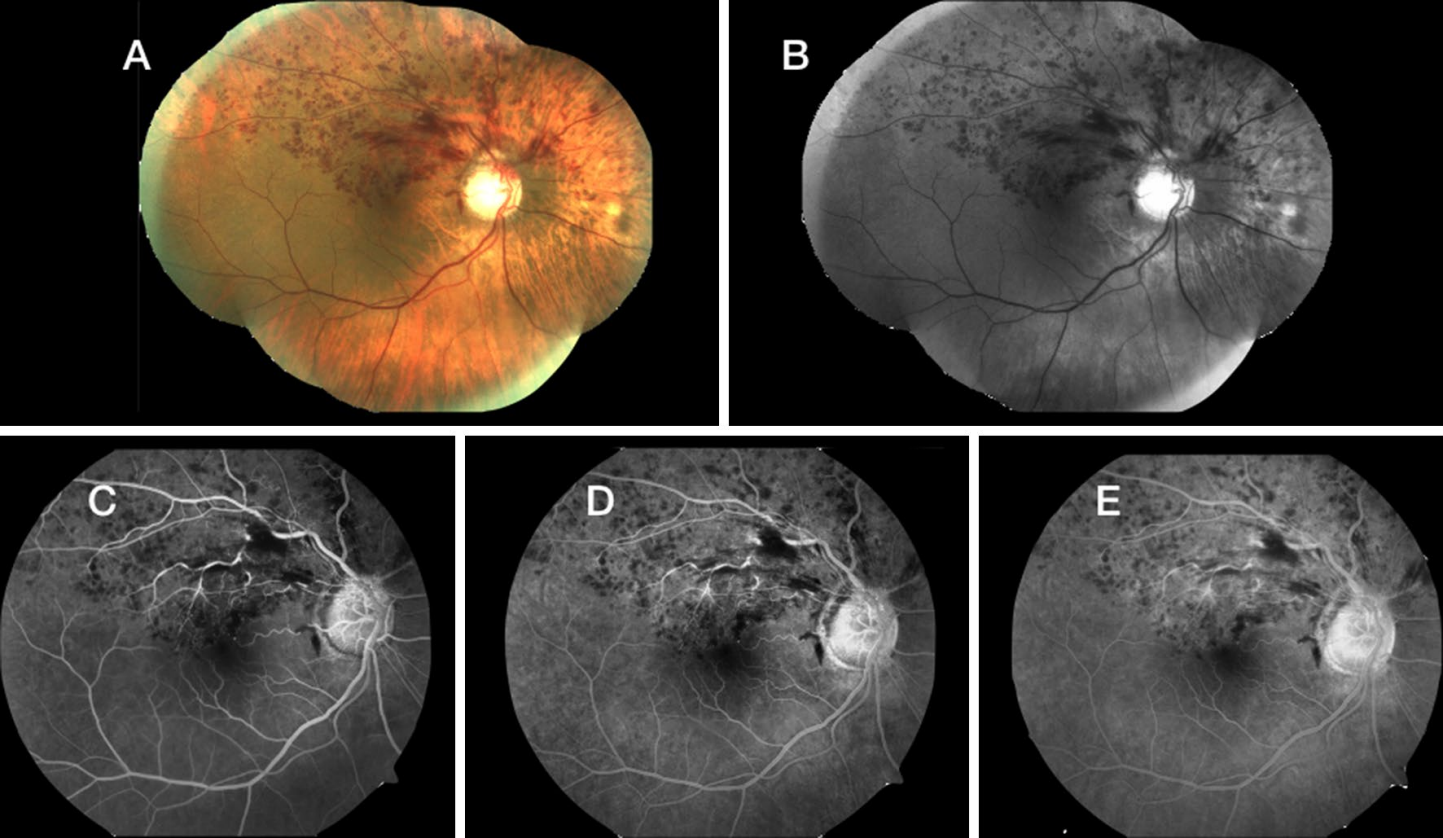
**A**–**E** A color fundus photograph and a series of fluorescein angiography show retinal hemorrhages in a patient with HRVO

A 43-year-old Caucasian man without comorbidities had HRVO. He presented with a painless decrease in VA in his right eye to 20/50 during 10 days of hospitalization to treat the COVID-19 infection. Biomicroscopy was unremarkable. Fundus examination of the right eye showed widespread hemorrhages and axonal congestion of the hemispheric superior retina (Fig. 1). The optic nerve appearance was suspicious for glaucoma (0.8 × 0.7), however glaucoma was not confirmed during his follow-up previous to the RVO episode. His visual field was normal, IOP of 13 mm Hg in both eyes without medication and central corneal thickness of 520 microns. OCT images of the right eye showed macular edema. He was treated with six anti-VEGF intravitreal injections. The final VA was 20/30.

## Group 3

Three patients (2 men, 1 woman; average age, 51.3 years; range, 27–64 years) had BRVO. None had severe COVID-19 requiring hospitalization. The retinal vein branch involved was superotemporal in two cases and inferotemporal in one case. The mean central foveal thickness at presentation was 441 microns.

One of these patients was a 36-year-old man diagnosed with BRVO in the right eye about 3 months after mild symptomatic COVID-19 confirmed by serologic testing (IgM-IgG rapid test). His VA was 20/30 in the right eye. He was treated with one intravitreal injection of bevacizumab (Avastin, Genentech Inc., South San Francisco, CA, USA) and due to the prompt clinical response and the VA improvement to 20/20 he opted to not continue with anti-angiogenic treatment.

A 64-year-old man was seen 7 days after the onset of COVID-19 symptoms. The best-corrected VA (BCVA) was 20/20 in the right eye and counting fingers in the left eye. The pupillary reflexes were normal, and biomicroscopy of the anterior and posterior segments did not reveal cells in either eye. Color fundus photography showed exudates and intraretinal hemorrhages in the posterior pole, including the macular area, and fluorescein angiography revealed hypo- and hyper-fluorescence on the topography of the macular area of the left eye, indicating retinal non-perfusion and vascular leakage, respectively. After the intravitreal bevacizumab injection, there was a marked reduction in the amount of macula edema in the left eye at 1-month follow-up at which time the VA was 20/200.

## Group 4

**Fig. 2:**
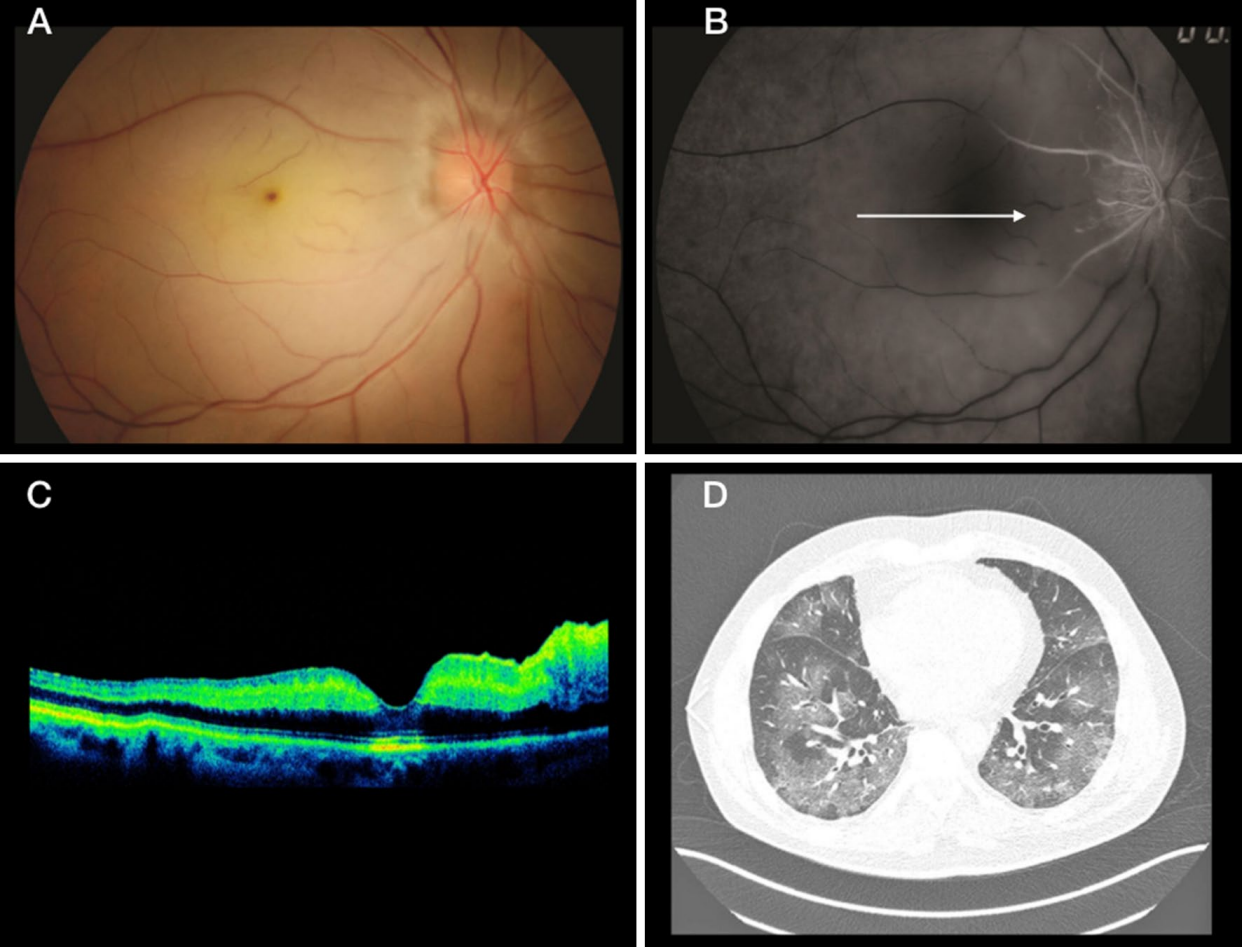
**A** Retinography of the right eye shows diffuse retinal paleness with a cherry-red spot in the macula. **B** Fluorescein angiography shows CRAO. **C** OCT image. **D** A chest nuclear magnetic resonance image. The arrow in B indicates occlusion of the cilioretinal artery

Two patients had CRAO. A healthy 47-year-old man and a 73-year-old man with systemic hypertension and diabetes were both hospitalized with severe COVID-19. Both had a similar history of painless visual loss to the level of light perception 7 days after COVID-19 was diagnosed. Fundus examination showed diffuse paleness and a cherry-red spot in the macula (Fig. 2). The work-up included neuroimaging, echocardiography, and angiographic imaging to evaluate the patient for carotid and cardiac disease, because potential systemic embolic sources did not show any relevant data. D-dimer was 40,4 ng/mL and C-reactive protein 8.02 mg/mL. No treatment was indicated in these cases and the VA remained unchanged at 2 months of follow-up.

The initial and final VAs and treatments are shown in Table 1.

**Table 1:** Distribution of vascular occlusion cases

n	Diagnosis	Gender	Age	Days until symptoms	VA i	VA f	Severity of COVID	Commorbility	Hospitalization	Treatment
1	CRVO	M	52	22	20/400	20/20	+	No	No	Anti VEGF
2		M	65	90	20/1000	20/400	+ +	No	No	Anti VEGF
3		F	37	45	20/20	20/20	+ +	No	No	No
4		M	46	50	20/40	20/20	+ +	No	No	Anti VEGF
5		F	27	7	20/60	20/20	+ +	No	No	Anti VEGF
6		M	36	55	20/20	20/20	+	No	No	No
7		M	41	45	20/40	20/30	+ + +	Dyslip	No	Anti VEGF
8		M	41	55	20/400	20/50	+ +	No	No	Anti VEGF
9	HMVO	M	43	7	20/50	20/30	+ + +	No	Yes	Anti VEGF
10	BRVO	F	54	7	20/25	20/20	+	No	No	Ketorolac d
11		M	36	90	20/30	20/20	+	No	No	Anti VEGF
12		M	64	7	CF	20/200	+ +	No	No	Anti VEGF
13	CRAO	M	73	7	LP	LP	+ + + +	DM + HBP	Yes	No
14		M	47	7	LP	LP	+ + + +	No	Yes	No

*CRVO* central retina vein occlusion, *HMVO* hemispheric vein occlusion, *BRVO* branch vein occlusion, *CRVO* central retina vein occlusion, *Days until symptoms* days after covid-19 infection until the onset of visual symptoms, *VA i* initial Visual acuity, *VA f* final Visual acuity, *Dyslip*: dyslipidemia, *DM*: Diabetes mellitus, *HBP* hight blood pressure, *Ketorolac d* drops

The National Institutes of Health (NIH) classified adults with SARS-Cov-2 infection into the following severity of illness categories: asymptomatic (0), mild illness with any of signs or symptoms, without dyspnea or abnormal chest imaging ( +), moderate illness lower respiratory disease or imaging and SpO2 > 94% (++), severe illness SatpO2 < 94%, respiratory rate > 30 breaths/min or lung infiltrates > 50% (+++) and critical illness: individual with respiratory failure, septic shock and/or multiple organ dysfunction (++ ++). Table 1

Most patients had no history of systemic hypertension or thrombotic events. Only one 41-year-old patient with CRVO used medication to treat dyslipidemia and hypertriglyceridemia, and one patient in the CRAO group had systemic hypertension and controlled diabetes mellitus.

## Discussion

The pathogenesis of COVID-19 remains poorly understood, although there is increasing evidence that an inflammatory cytokine storm and viral evasion of cellular immune responses play a fundamental role in disease severity and progression [7]. COVID-19 may predispose patients to thromboembolic events, both in the arterial and venous circulations, due to excessive inflammation, endothelial dysfunction, platelet activation, and stasis [8]. The cytokine cascade seen in COVID-19 resulted from the inflammatory response to severe infection and also to the direct effect of SARS-CoV-2 on the endothelium. The inflammatory response and direct viral effect contribute to the disease severity, resulting in complications such as venous thromboembolism and mortality associated with COVID-19.

Endothelial alterations and endotheliitis are important determinants of microvascular dysfunction, which disrupts the vascular balance toward vasoconstriction, ischemia, tissue edema, and a procoagulant state. Concurrent with the altered coagulation, an exacerbated proinflammatory cytokine response has been observed in patients with COVID-19 (C-reactive protein, ferritin, interleukin [IL]-2, IL-6, IL-7, IL-10, interferon-gamma inducible protein 10, tumor necrosis factor-α, etc.), although the cause of this cytokine storm remains unclear. The SARS-CoV-2 cytokine storm precipitates the onset of a systemic inflammatory response syndrome, resulting in activation of the coagulation cascade that induces to a hypercoagulable state. However, whether the virus directly activates the coagulation cascade or whether this is the result of local or systemic inflammation is not completely understood. These findings are consistent with the close connection between thrombosis and inflammation [9].

The severity of the retinal vascular occlusive events in the present case series varied from mild retinal vein occlusion with a BCVA exceeding 20/60 in eight cases, severe retinal vein occlusion with a BCVA worse than 20/60 in three cases, and retinal arterial occlusion in 2 cases. Individual factors or the severity of the COVID-19 infection might explain the variable presentations of the retinal vascular phenomenon.

The occurrence of CRVO in adults under 60 years of age has been reported to be considerably less common than in older patients. Systemic hypertension was the most prevalent risk factor for CRVO in young patients, followed by diabetes mellitus and hyperlipidemia [10]. Nevertheless, hypercoagulability often is reported as a significant risk factor for CRVO in younger individuals, in particular, deficiencies of anticoagulant protein C, S, and antithrombin III and activated protein C resistance [11]. This was the case of patient 5, from group 1, a 27 years old healthy female, with no use of anticonception pills, who had a CRVO with high seric levels of D-dimers and PCR following COVID-19 infection. Subsequent lab work up with the hematologist did not find any other possible cause of the significant thrombotic event.

In the current case series, the mean patient age was 47.97 years and most patients (85.72%) had no previous history of systemic hypertension or thrombotic events. The only common patient background was the previous COVID-19 infection. It is not possible to identify a direct cause and effect of the viral infection and retinal vascular occlusion. However, understanding the pathophysiology of the SARS CoV-2 infection, the COVID-19 can be viewed as a prothrombotic disease and may justify this retinal vascular disease in such young patients.

Two major possible mechanisms might explain vascular damage in COVID-19 disease, first a pseudo-vasculitis state as a result of a viral infiltration of the endothelial cells, and second, a hypercoagulable condition, characterized by a disseminated intravascular coagulation-like scenario. These mechanisms might explain the association between the potential impact of COVID-19 on the retinal vascular circulation and the occurrence of a retinal vascular disease as seen in the current cases.

The effect on the retinal circulation in patients with COVID-19 has been suggested previously and reinforces the idea that the effect is not coincidental but rather a disease consequence. The presentation of unilateral CRVO in our young, healthy patients without any identifiable hypercoagulability risk factors shortly after recovery from COVID-19, may imply that CRVO or CRAO in our patients was related to the thromboembolic state associated with the SARS-CoV-2 infection [12].

Most patients experience a non-arteritic CRAO. In non-arteritic CRAO, the retinal artery is occluded from a platelet–fibrin thrombus or embolism from an atherosclerotic lesion or hypercoagulable state such as with COVID-19 [13]. Some studies have shown an incidence of venous and arterial thrombotic events in more than 30% of COVID 19 patients, with venous thromboembolic events being the most common (27%) [6]. Patients with moderate to severe infection are more likely to have COVID-19- associated coagulopathy (CAC) and these thrombotic complications can occur even in late stages of the disease or early recovery. The most common pattern of coagulopathy observed in patients hospitalized with COVID-19 is characterized by elevations in fibrinogen and D-dimer levels. This correlates with a parallel rise in markers of inflammation [9].

Al-Moujahed et al. [14] published in 2022, a retrospective cohort study of patients with a new diagnosis in two time periods: pre–COVID-19 period and the COVID-19 period. They concluded the percentage of new cases of RAO and RVO remained stable for the majority of the COVID-19 period. There was an increase in these percentages during the first few months of the COVID-19 pandemic, particularly for CRAO, CRVO, and BRAO.

This study has some limitation in that it is an uncontrolled case series, the time until symptom onset varied considerably (range, 7–90 days), and we did not compare the incidence of the retinal vascular occlusion to a similar timeframe (2018/2019), but it is a first step for future confirmatory controlled studies to show an association between COVID-19 infection and retinal vascular occlusion.

## Conclusions

This is the largest case series thus far that describes 14 patients with retinal vascular occlusion after a COVID-19 infection. It well known that patients with COVID-19 are at risk of presenting with venous and arterial thrombotic events, and these thrombotic events are actively involved in the disease pathophysiology. The three main factors involved in the pathogenesis of coagulopathy in patients with COVID-19 are endotheliitis, which causes mechanical problems through vasoconstriction; hyper-inflammation and a cytokine storm that activate clotting factors; and stasis and hypoxia that also activate coagulation mechanisms. Clinicians and ophthalmologists must be aware of the effect of COVID-19 beyond the involvement of the ocular surface that occurs in the intermediate stages of COVID-19 and that there may be an increased incidence in the number of patients with ocular vascular diseases due to the hyper-inflammatory and hypercoagulable state triggered by a SARS-CoV-2 infection.

## Data Availability

Not applicable.

## Abbreviations

COVID-19: Coronavirus disease
SARS-CoV-2: Severe acute respiratory syndrome coronavirus 2
RT-PCR: Reverse transcriptase-polymerase chain reaction
CRVO: Central retinal vein occlusion
HRVO: Hemispheric retinal vein occlusion
BRVO: Branch retinal vein occlusion
CRAO: Central retinal arterial occlusion
BCVA: Best-corrected visual acuity
VEGF: Vascular endothelial growth factor
OCT: Optical coherence tomography; resonance magnetic nuclear
